# Repositioning of the Severe Prolapsed Silicone Tubes after Bicanalicular Nasal Intubation: A Novel Technique

**DOI:** 10.1155/2021/6669717

**Published:** 2021-03-06

**Authors:** Jinjing He, Jingwen Gong, Qingqing Zheng, Jin Jiang

**Affiliations:** Department of Ophthalmology, Zhejiang Provincial People's Hospital, People's Hospital of Hangzhou Medical College, Hangzhou, Zhejiang 310014, China

## Abstract

**Background:**

Bicanalicular nasal intubation is widely used in lacrimal drainage system surgery. Its common complication is lateral displacement or spontaneous prolapse. When the distal part of the silicone tubes cannot be seen in the nose endoscopically, either repositioning or removal could be a challenge. We developed a simple technique to reposition the severe prolapsed silicone tubes.

**Method:**

This retrospective study included 6 patients with severe prolapsed silicone tubes who had undergone bicanalicular nasal intubation between January 2017 and December 2019. We used a memory wire probe to pull a nylon suture through the lacrimal passage retrograde. Then, the nylon suture was cut into two lines. One line was coiled to the prolapsed tube and tied to another line. This nylon turned into a “lasso” to capture the silicone tube and then lock its knot. By pulling the nylon suture, the severe prolapsed silicone tube was repositioned to the nasal cavity.

**Results:**

Using this technique, we successfully repositioned severe prolapsed silicone tubes without any complication in 6 cases.

**Conclusions:**

Silicone tube reposition guiding by using a memory wire probe is an optional technique in the treatment of prolapse of silicone tubes, particularly if the distal part of the silicon tube was embedded in the lacrimal sac and cannot be seen in the nose by endoscopy. It is a feasible, minimally invasive, safe, and effective method, avoiding premature tube removal.

## 1. Introduction

Epiphora is a socially and functionally bothersome symptom. Various surgical techniques for lacrimal system surgery have been described in previous study. During these procedures, silicone tubes are widely used to maintain the patency of the newly created fistula and to prevent scarring and stenosis of the ostium [1–3]. Bicanalicular nasal intubation with the silicone tube is one of the widely performed treatments. However, lateral prolapse of the silicone tube is one of its most common complications [4, 5]. It may result in poor cosmesis, corneal and conjunctival irritation, and corneal erosion. Concerning for these complications, reposition of the tubes is required. Various methods to reposition the tubes have been reported. When the lateral prolapse is slight, we can simply push them back into the canaliculi or pull their ends down inside the nose under with or without endoscopic guidance [5]. If the tubes cannot be repositioned, they should be removed [6, 7]. If the lateral prolapse of the silicone tube occurs soon after surgery, a loss of the silicone tube calls for a necessity to reintubate the system. However, in some cases, severe lateral prolapses of the silicone tube occur in which the distal part of the silicone tube is deep within the nasolacrimal duct or embedded in the lacrimal sac. The silicone tubes could not be seen in the nose by endoscopy. In this case, either repositioning or removal could be a challenge. To solve the problem, we developed a new technique to reposition the prolapsed silicone tubes avoiding premature silicone tube loss.

## 2. Subjects and Methods

### 2.1. Patients

**Figure 1:**
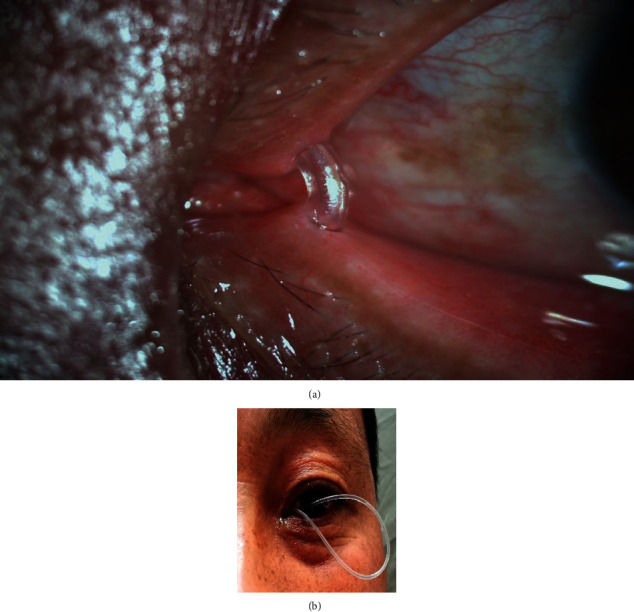
(a) After the bicanalicular-nasal silicone tube is placed by standard methods, little segment of the silicone tube is left to be exposed at the medial canthus. (b) Severe lateral prolapse of the silicone tube at the medial canthus. The loop is lying in front of the cornea, resulting in poor cosmesis, corneal and conjunctival irritation, and corneal erosion if left.

From January 2017 to December 2019, we experienced 6 cases of stenosis of the lacrimal canaliculus, treating with bicanalicular nasal intubation, in which the silicon tube was pulled out at the medial canthus by inattention. The prolapse was so severe (Figure 1) that the distal knots of the silicon tube were not seen in the nose by endoscopy. The timings of the prolapse were all less than 50 days. It was too early to remove the silicon tube. So, we tried to reposition the prolapsed tubes to avoid premature tube removal. All the surgeries were performed by the same surgeon (JJ) under local anaesthesia. The patients were completely informed of the potential risks before the surgery. Written informed consent was obtained from all the patients. This study was consistent with the Declaration of Helsinki and was approved by the ethics committee of Zhejiang Provincial People's Hospital before applying this surgical procedure clinically.

### 2.2. Special Instrument

**Figure 2:**
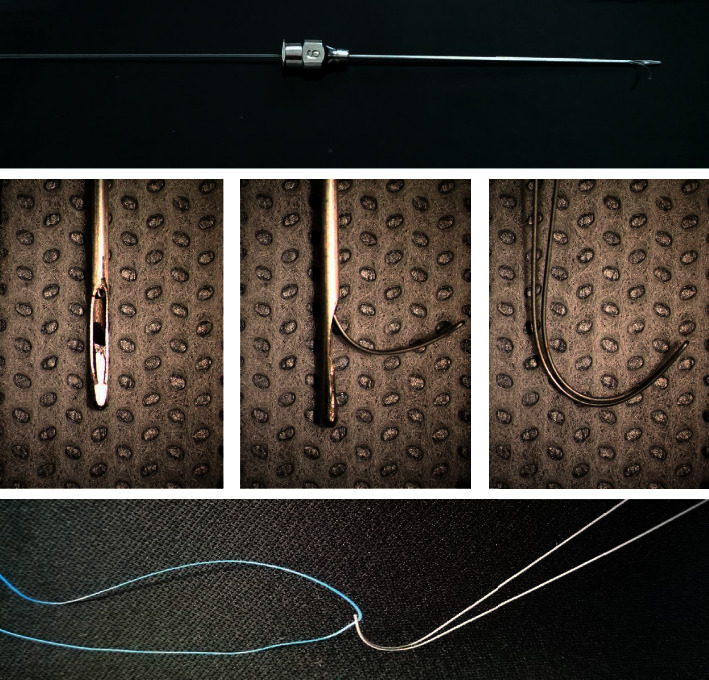
Memory wire probe consists of a stainless steel syringe needle probe with its memory wire inside. The syringe needle probe has a closed round tip and a side port which allows the memory wire in and out. The memory wire is made of two strands with a joint head, allowing it to clip the nylon suture.

The special instrument used for repositioning of the prolapsed tubes was the memory wire probe (Map into medical technology Co., Ltd., Hangzhou, China). It consists of a stainless steel syringe needle probe with the memory wire inside (Figure 2). The probe has a closed round tip and a side port which allows the memory wire in and out. The direction of the side port is the same with carved number “9” on the syringe adapter. The memory wire is made of titanium-nickel alloy and made into two strands with a joint head (Figure 2). It has good deformation ability, making into a shape of a curved head and straight body. Its shape conforms to the characteristics of the inferior nasal meatus. So, when the tail of the memory wire is pushed, its head will go out of the nostril following the inferior nasal meatus. The flexibility also allows it to reduce injury to the nasal mucosa. Moreover, it has the ability to recover from deformation, allowing it to clip the nylon suture and to be pulled back into the probe.

### 2.3. Reposition Process

**Figure 3:**
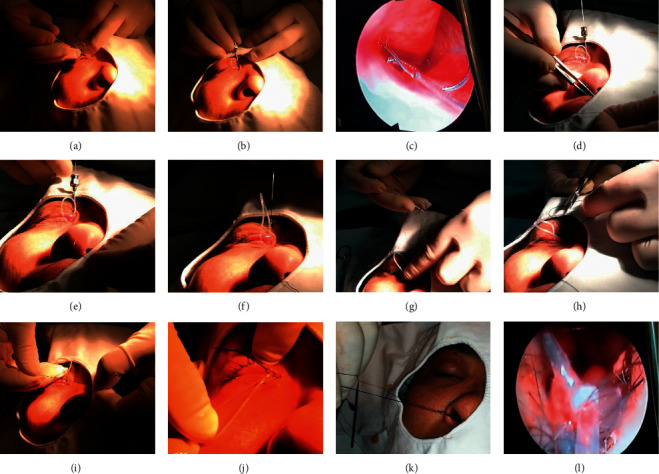
The surgical maneuvers for repositioning the silicone tube. (a) A memory wire probe is gently introduced through the punctum and passed down the nasolacrimal duct to the nasal cavity. (b) The probe is rotated to place carved number “9” face up. The tail of the memory wire is gently pushed, making its head out through the probe. (c) A video endoscope is put into the nasal cavity to ensure that the probe is not coming out of any false passages, hooking out the memory wire. (d) and (e) A 5-0 nylon suture is threaded through the memory wire's head. (f) The memory wire is withdrawn into the probe. Then, the probe is pulled, together with the nylon suture, through the lacrimal passage retrograde until it is out of the punctum. (g) The probe and the memory wire are removed. (h) The 5-0 nylon suture is cut into two lines from the middle. (i) One line of the nylon is coiled to the silicone tube and tied to another line. (j) This turns into a “lasso” to capture the silicone tube. (k) Gentle traction is kept on the distal end of the nylon suture, and the “lasso” slides down from the punctum to the dacryocyst around the silicone tube, pulling the silicone tube into its proper position. (l) Nasal endoscope shows the reposition of the silicone tube and its knot.

The steps of repositioning the tubes are described as follows (Figure 3):

- A memory wire probe is gently introduced through the punctum and passed down the nasolacrimal duct to the nasal cavity. In this process, a video endoscope (Storz Tricam SL Endoscope, Tuttlingen, Germany) is put into the nasal cavity to ensure that the probe is not coming out of any false passages.
- Rotate the probe to make the side engraved with “9” face up. The tail of the memory wire is pushed gently, making its head out through the probe, until the memory wire's head is out of the nasal cavity.
- A 5-0 nylon suture is threaded through the memory wire's head. The memory wire is withdrawn into the probe. Then, the probe is pulled, together with the nylon suture, through the lacrimal passage retrograde until it is out of the punctum.
- At the medial canthus, this 5-0 nylon suture is cut from the middle into two lines. One line of the nylon is coiled to the silicone tube and tied to another line. This turns into a “lasso” to capture the silicone tube.
- One hand of the surgeon fixes the silicone tube loop, and the other hand pulls the distal end of the nylon suture carefully from the nostril. The “lasso” slides down from the punctum to the dacryocyst around the silicone tube until it is locked by the knots of the silicone tube.
- Gentle traction is kept on the distal end of the nylon suture. Then, the silicone tube is drawn into its proper position. Finally, the nylon suture is removed.

**Figure 4:**
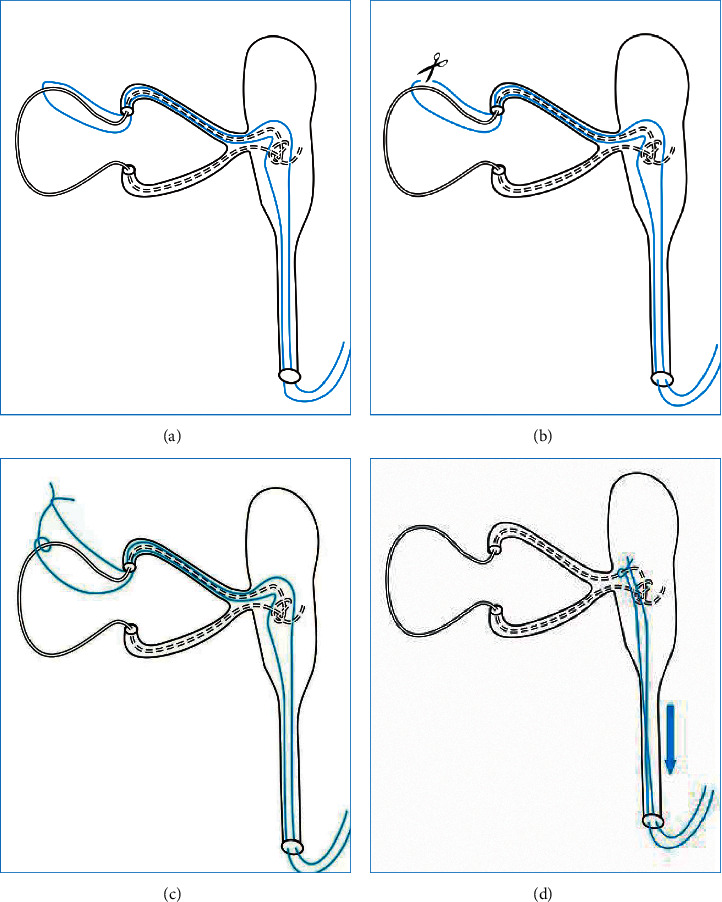
Schematic diagram of repositioning prolapsed silicone tubes. (a) Severe lateral prolapse of the silicone tube at the medial canthus. The distal part of the silicone tube is deep within the nasolacrimal duct or embedded in the lacrimal sac. The middle of a 5-0 nylon suture is pulled through the lacrimal passage retrograde by withdrawing the memory wire probe. (b) The 5-0 nylon suture is cut into two lines from the middle. (c) One line of the nylon is coiled to the silicone tube and tied to another line. This turns into a “lasso” to capture the silicone tube. (d) The distal end of the nylon suture is pulled from the nostril, and the “lasso” slides down from the punctum to the dacryocyst around the silicone tube until it is locked by the knots of the silicone tube.

Since the silicone tube embedded in the lacrimal sac was invisible, we have drawn a schematic diagram, as shown in Figure 4.

## 3. Results

Six patients, 4 men and 2 women, had a mean age of 46.7 ± 5.0 years (range: 40–56 years). The average interval between the onset of symptoms of epiphora and the surgery was 3.3 ± 1.2 years (range: 2–5 years). Mean follow-up duration was 10.0 ± 1.6 months. All patients completed 6 months of follow-up. The whole surgery process took about 20 minutes. The patients had only mild discomfort. Silicone tube reposition guiding by using the memory wire probe was performed successfully in all patients without any complications such as bleeding or silicone tube damage. The patients retained the silicone tube for six months without recurrence of the prolapse. After the silicone tube was removed, the canaliculi were patent, and the patients had no epiphora.

## 4. Discussion

Lateral prolapse of the silicone tube is one of the most common complications associated with silicone intubation. A literature review performed by Brookes and Olver [5] cited that tube prolapse occurs in up to 17.5% patients after nasolacrimal duct intubation and 14% after dacryocystorhinostomy (DCR). Most of them can be repositioned by feeding them back down into the canaliculi or pulling their ends down inside the nose under endoscopic guidance. The situation that the distal part of the silicone tube is deep within the nasolacrimal duct or embedded in the lacrimal sac is rare but knotty. This usually occurs as a result of the patient wiping their eye with a finger or handkerchief by inattention. Under the panic of catching the silicone tube at the medial canthus, some patients pulled it excessively until the knot embedded in the lacrimal sac.

Several methods have been proposed to deal with this situation. Kathuria and Harvey cut the loop and pulled the knots out of the punctum retrogradely [8]. This method is now considered to be not advisable because the knots may damage the proximal part of the lacrimal apparatus [9, 10] such as the canaliculus or punctum. Cakmak cut the loop and attached the cut end of the silicone tube to a prolene suture, which had previously been withdrawn from the nose. By pulling the prolene suture, the tube could be removed through the nasolacrimal duct and out of the nose [7]. Yeh et al. chose to perform DCR and explore the sac to remove the persistent prolapsed tubes [11]. All these methods result in a loss of the silicone tube and may require another bicanalicular nasal intubation if a longer period of intubation is needed. According to the latest technology, endoscopic sinus surgery with computer-assisted navigation may be helpful to deal with this situation [12].

In the present retrospective study, we demonstrated a new technique to reposition the prolapsed tubes. The study population was small because the incidence of tube prolapse is rare. However, the results of our study show that silicone tube reposition guiding by using the memory wire probe was simple and effective. The whole surgery process took about 20 minutes. It can be done in the outpatient clinic without hospitalization. No extra expensive instrument is needed. The memory wire probe is a lacrimal probe with the memory wire inside. The use is similar with the ordinary lacrimal probe, requiring no extra learning. The nylon suture can be replaced by any kind of line which is smooth and strong enough to make the “lasso” slide down around the silicone tube. Moreover, our technique can reposition the prolapsed silicone tubes instead of removing them, avoiding the possibility of a second bicanalicular nasal intubation and additional financial burdens. This technique is feasible, minimally invasive, safe, and effective, avoiding premature tube removal. It could be considered as an optional technique to deal with severe prolapsed silicone tubes after bicanalicular nasal intubation.

## Data Availability

The data used to support the findings of this study are included within the article.

## Abbreviations

DCR: Dacryocystorhinostomy.
